# Biochemical Changes in Adult Male Gamers During Prolonged Gaming: Pilot Study

**DOI:** 10.2196/46570

**Published:** 2024-07-08

**Authors:** Kasper Bygum Krarup, Johannes Riis, Morten Mørk, Hien Thi Thu Nguyen, Inge Søkilde Pedersen, Søren Risom Kristensen, Aase Handberg, Henrik Bygum Krarup

**Affiliations:** 1 Department of Geriatrics Aalborg University Hospital Aalborg Denmark; 2 Department of Clinical Biochemistry Aalborg University Hospital Aalborg Denmark; 3 Department of Molecular Diagnostics Aalborg University Hospital Aalborg Denmark; 4 Department of Clinical Medicine Aalborg University Aalborg Denmark

**Keywords:** long gaming sessions, local area network party, biochemistry, cortisol, glucose, gaming, biochemical, blood sample, hematology, hematological, games, gamers, hemoglobin, adults, males, men, blood

## Abstract

**Background:**

Gaming has become an integrated part of life for children and adults worldwide. Previous studies on the impact of gaming on biochemical parameters have primarily addressed the acute effects of gaming. The literature is limited, and the study designs are very diverse. The parameters that have been investigated most thoroughly are blood glucose and cortisol.

**Objective:**

This exploratory study is the first to investigate the effects of long gaming sessions on the biochemical parameters of healthy male adults. The extensive testing allowed us to observe short-term changes (within 6 hours), long-term changes during the duration of the gaming sessions, and follow-up after 1 week to determine whether any changes were longer lasting.

**Methods:**

In total, 9 experienced gamers completed 2 back-to-back 18-hour gaming sessions interspersed with a 6-hour rest period. All participants adhered to a structured sleep pattern due to daytime employment or attending university. Blood, saliva, and urine samples were collected from the participants every 6 hours. Linear mixed-effect models were used to analyze the repeated-measures data accumulated during the study. A total of 51 biochemical parameters were investigated.

**Results:**

In total, 12 of the 51 biochemical parameters significantly changed during the study: alkaline phosphatase, aspartate aminotransferase, bilirubin, chloride, creatinine, glucose, hemoglobin, immature reticulocyte fraction, lactate, methemoglobin, sodium, and thrombocytes. All changes were within the normal range. The mean glucose level of the participants was 4.39 (SD 0.07) mmol/L at baseline, which increased significantly by 0.24 (SD 0.07) mmol/L per 6 hours during the first period and by 0.38 (SD 0.07) mmol/L per 6 hours in the second period (*P*<.001). The glucose levels during the second session increased even though the participants had little energy intake. Cortisol levels did not change significantly, although the cortisol pattern deviated from the typical circadian rhythm. During both gaming sessions, we observed increasing cortisol levels from 6 AM until noon. The participants were relatively dehydrated at the start of the study. The patients were asked to fast before the first blood sampling. Within the first 6 hours of the study, the participants rehydrated, followed by relative dehydration during the remainder of the study. This pattern was identified using the following parameters: albumin, creatinine, hemoglobin, erythrocytes, potassium, and platelets.

**Conclusions:**

This study is the first of its kind, and many of the analyses in the study yielded novel results. The study was designed to emulate the behavior of gamers during the weekend and other long gaming sessions. At this point, we are not able to determine the difference between the effects of gaming and behavior during gaming. Regardless, the results of this study suggest that healthy gamers can partake in long gaming sessions, with ample amounts of unhealthy foods and little rest, without acute impacts on health.

## Introduction

Video games have become a favorite pastime among children and adolescents. In the United States, 99% and 94% of boys and girls, respectively, play video games [1]. Video games are part of a larger category of sedentary activities linked to health issues such as physical inactivity, overeating, obesity, and diabetes [2-6]. Collegiate-level gamers have a comparable BMI to their nongaming peers but are less active with a higher body fat percentage, lower lean body mass, and lower bone mineral content [7]. This is particularly troublesome because studies report that children spend as much as 7-11 hours daily engaged in screen-based activities after school [8]. In addition, gaming has been linked to overeating and overconsumption of soft drinks as well as adverse health behaviors [9-12]. In a recent review, the authors found that while gaming may increase energy expenditure above baseline levels, gaming does not constitute physical activity. Energy expenditure may increase, but the activity level is comparable to that of standing or walking [13].

Previously published data from this study showed that the participants ingested an excessive number of calories from both food and drink [14]. During the study, the participants ingested an average of 6160 kcal from food and 1844 kcal from liquid sources. Additionally, the participants consumed 1354 mg of caffeine on average during the same period [14].

Previous studies of the impact of gaming on biochemical parameters have primarily addressed the acute effects of gaming. The literature is limited, and the study designs are very diverse. The parameters that have been investigated most thoroughly are blood glucose and cortisol. For blood glucose levels, gaming does not appear to increase glucose levels within the first 20 minutes of a gaming session [15,16]. Chaput et al [15] reported that gaming increased blood glucose levels after 40 minutes of a 60-minute gaming session.

Cortisol has been used as a marker of both physiological and mental stress during gaming [17-19]. Gaming may affect cortisol levels, but the nature of this relationship has yet to be elucidated. Oxford et al [17] reported that cortisol levels increase acutely when gamers compete against friends. The authors suggested that this was typical of male-male competition behavior [17].

While biochemical markers such as blood glucose and cortisol levels have been investigated during the last 2 decades, many biochemical markers pertaining to acute changes in the health of the human body have yet to be investigated. In this study, we aimed to investigate a broad array of biochemical markers to assess homeostasis, lipid metabolism, internal organ function, hematological balance, acid-base balance, and blood gases during long gaming sessions. This is the first time that most of the included parameters (except for glucose and cortisol) were investigated in gamers and during long gaming sessions. As such, we did not know what to expect over the course of the study.

According to a recent literature review, knowledge of the effects of long video gaming sessions is minimal [13]. This exploratory study is the first of its kind in gaming. This is true for both the length of the gaming sessions, the physical setup, and the extensive testing.

A limitation of this study was the relatively small number of participants. This study was designed to realistically emulate the gaming behavior of young adults in a controlled setting in a hospital dining hall and an adjourning meeting room. The study was conducted as a local area network party for practical reasons. Four laboratory technicians worked at all times to sample, prepare, and analyze blood samples. A doctor and 2 investigators were also present throughout the gaming sessions. We tried to overcome the artificial situation of the event by discussing the setup with the participants before the study to create the most real-life–like experience (video clip [20]). The participants were asked to consume food and drink according to their wishes and what they would habitually consume [14]. The extensive testing in the study allowed us to observe short-term changes (within 6 hours), long-term changes during the duration of the gaming sessions, and follow-up after 1 week to determine whether any changes were longer lasting. The aim of this study was to investigate the effects of long gaming sessions on the biochemical parameters of healthy male adults.

## Methods

### Participants and Intervention

We have previously presented how the study was conducted and the physiological response to long gaming sessions [14]. In this paper, the results of an extensive collection of biochemical data are presented. We have adhered to the CONSORT (Consolidated Standards of Reporting Trials) statement regarding pilot and feasibility trials [21]. A CONSORT checklist can be found in Multimedia Appendix 1.

In brief, 9 healthy male participants older than 18 years of age with vast gaming experience were enrolled. The mean age of the participants was 25.8 (SD 2.6) years, and the mean BMI was 24.8 (SD 2.9) years. All participants were either full-time students or employees [14]. According to the protocol (EudraCT 2019-004091-19), the plan was to enroll 6-9 gamers, with at least 6 gamers engaging in gaming for 48 hours. Participants were enrolled after they had called for participants from local e-sports clubs through e-sports instructors, online message boards, and word of mouth. The participants were recruited into 2 teams of 4 and 5 members.

The intervention consisted of two 18-hour gaming sessions interspersed with a 6-hour break. During the break, the participants had approximately 4 hours of sleep. After the last gaming period, all participants underwent both physiological and biochemical tests.

Throughout the intervention, the participants had ad libitum access to food and drink. Before the study, participants provided lists of their preferred snacks and drinks. An assortment of chips, candies, cookies, buns, cold cuts, cereal, and fruit was available throughout the study. Additionally, an evening meal was provided at 6 PM during both gaming sessions: pizza on the first evening and hamburgers on the second evening. Participants had access to tap water, soda, energy drinks, coffee, milk, and chocolate milk.

The participants were instructed to avoid strenuous physical labor, cardiovascular exercise, alcohol, and junk food for 7 days before the study. Additionally, on the day of the study, the participants were instructed to stop food intake at noon and only drink water in case of thirst.

During the study, the participants were not restricted in any way regarding gaming. Specifically, all types of games across all genres and platforms were allowed. One participant brought his PlayStation, and as a new soccer game had just been released, participants played matches in teams or head-to-head. At other times, the participants played alone in certain games or played on the web with and against players who were not participants in the study. Especially during the last 18 hours of the study, the participants played first-person shooter games against each other on teams.

### Participant Involvement

After the protocol was approved by the local ethics committee in February 2018, in total, 2 meetings were held with potential participants and local e-sports instructors, discussing the content of the protocol. In particular, food monitoring and blood sampling methods were changed in accordance with the wishes of the participants.

The participants suggested a “food diary” to monitor their caloric intake. This suggestion was incorporated into the protocol. First, it was suggested by the research team that a venous catheter be inserted for blood sampling throughout the study. Instead, the participants opted for multiple venipunctures. The changes suggested at the meetings were approved by the local ethics committee in an amendment before the study started.

### Ethical Considerations

The North Denmark Region Committee approved the study protocol on Health Research Ethics (N-20180011; EudraCT 2019-004091). Each participant provided informed consent in writing twice, 2 weeks prior to the study and again on the day of the study. The study used a standardized consent form that stated that participation was voluntary and that participants could withdraw from the study at any time without reason or consequence. Primary consent was obtained after approval for the secondary analysis. The data were deidentified. Except for travel expenses, participants were not compensated for their participation.

### Blood Sampling and Processing

When the participants arrived at the laboratory, baseline samples were collected, including a venous blood sample, a urine sample, and a saliva swab. Blood, urine, and saliva were collected from the participants every 6 hours. After the blood was drawn by venous puncture, it was taken to the laboratory. Biochemical analyses were performed immediately using an ABL800 FLEX Blood Gas Analyzer (Radiometer); other samples were centrifuged (at 3000 rpm/1875×*g* for 10 minutes), and the plasma was frozen at –80 °C for later analysis. Blood samples were analyzed using a Cobas 8000 Modular Analyzer (Roche Applied Science) and a Sysmex XN-9000 Hematology Analyzer (Sysmex Europe, GmbH). In total, 18 parameters were analyzed on the Cobas 8000 (1 parameter is only presented in Multimedia Appendices 2 and 3), 22 parameters were analyzed on the Sysmex XN-9000 (16 parameters are only presented in Multimedia Appendices 2 and 3), and 18 parameters were analyzed using the ABL800 (11 parameters are only presented in Multimedia Appendices 2 and 3). An overview of the analyses by apparatus can be found in Multimedia Appendix 2. The complete summary of the results is provided in Multimedia Appendix 3. A total of 8 parameters were analyzed twice between the 3 machines (calcium [Ca], glucose, lactate, potassium [K], sodium [Na], bilirubin, creatinine, and hemoglobin [Hb]). All the tests were performed in accordance with the manufacturer’s instructions in a nationally accredited biochemistry laboratory (Department of Clinical Biochemistry, Aalborg University Hospital).

The following 30 parameters were measured and are presented in the Results section: glucose (mmol/L), lactate (mmol/L), cortisol (nmol/L), Ca (mmol/L), albumin-corrected calcium (mmol/L), K (mmol/L), Na (mmol/L), and chloride (Cl, mmol/L) were measured to assess homeostasis. Low-density lipoprotein (LDL) cholesterol (mmol/L), high-density lipoprotein cholesterol (mmol/L), total cholesterol (mmol/L), and triglyceride (TG, mmol/L) levels were measured to assess lipid metabolism. Alanine aminotransferase (ALT, U/L), albumin (g/L), alkaline phosphatase (ALP, U/L), bilirubin (µmol/L), creatinine (μmol/L), C-reactive protein (CRP, mg/L), and ferritin (µg/L) were measured to assess organ-specific markers. sO_2_ (%), pCO_2_ (kPa), pO_2_ (kPa), pH, and standard bicarbonate concentration (mmol/L) were measured to assess acid-base balance and blood gases. Erythrocytes (10^12^/L), erythrocyte volume fraction (EVF), Hb (mmol/L), mean cell Hb (10^–15^, SE 0.0018 mol), mean cell volume (10^–15^/L, SE 0.088/L), and platelets (10^9^/L) were measured to assess the hematological markers.

### Statistical Analyses

Linear mixed-effects models were used to analyze participants’ absolute changes in biochemical parameters throughout the study. This modeling method is a standard extension of linear regression models that controls for the random effects introduced by having paired data and is often used when analyzing studies with repeated measurements. We performed separate univariate analyses for each change in each biochemical parameter; time was the fixed effect, and participants were the random effect. Additionally, we included an interaction term between time and period. This gave separate results for the first and second gaming periods.

We used a likelihood ratio test to test the statistical significance of the linear mixed effects models compared to a basic model, which included only random effects and thereby assumed no development over time. In total, 51 markers were analyzed (58 markers in total and 7 duplicates), and Bonferroni correction was applied by multiplying α (originally .05) by the number of tests. Statistical significance was set at α=.05 before Bonferroni correction [22]. The assumptions of linear mixed-effects models were tested and fulfilled in all the analyses. Linearity and homoscedasticity were assessed by inspecting residual plots. The normality of the residuals was assessed by inspecting histograms and quantile-quantile plots. Given that this was an exploratory study, outliers were not removed. Due to the sample size and number of tests, we did not perform post hoc tests comparing the data at individual time points.

**Figure 1:**
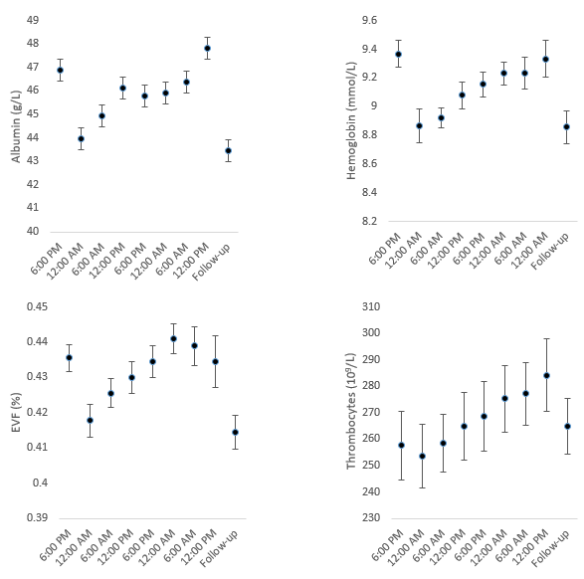
Dehydration. (A) Albumin, (B) hemoglobin, (C) erythrocyte volume fraction (EVF), and (D) platelets.

**Figure 2:**
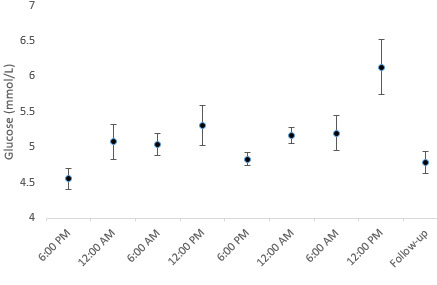
Glucose development over time.

**Figure 3:**
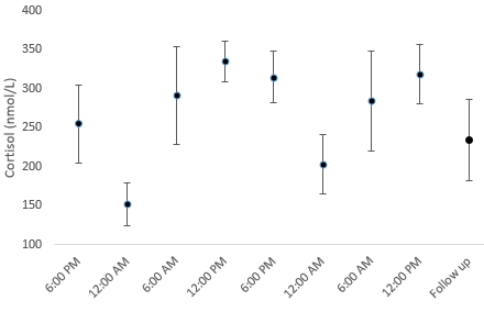
Cortisol development over time.

**Figure 4:**
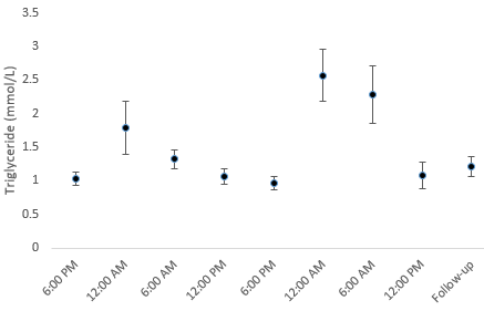
Triglyceride development over time.

Graphs illustrating changes over time were made for all variables, consisting of average values among participants for each sampling point, together with SEs presented as error bars from the mean. In addition, selected graphs were included to illustrate developments over time for specific parameters (Figures 1-4) that were not necessarily reflected in the linear mixed-effects models.

One participant’s LDL cholesterol level decreased 3-fold below the minimum measuring range. The missing values were substituted by using the previous lowest value of the participant [23]. All the statistical analyses were performed using Microsoft Excel 2013 (Microsoft Corp) and RStudio (version 1.1.383; Posit, PBC). Linear mixed-effects models were generated using the *lme4* package [24].

## Results

### Overview

The linear mixed-effects models produced results with a mean baseline value at the start of the study and coefficients of change for the first and second gaming periods. Several parameters, such as albumin, Hb, EVF, and the platelet count, exhibited a pattern of high initial values, followed by a decrease after 6 hours and a continuous increase during the rest of the gaming period (Figure 1). The complete list of results of all analyses is available in Multimedia Appendix 3.

### Homeostasis

The mean glucose level of the participants was 4.39 (SD 0.07) mmol/L at baseline, which increased significantly by 0.24 (SD 0.07) mmol/L per 6 hours during the first period and by 0.38 (SD 0.07) mmol/L per 6 hours in the second period (Figure 2). The mean Na level was 141.9 (SE 0.12) mmol/L at baseline and decreased by 0.31 (SD 0.1) mmol/L per 6 hours during the second period. There were no changes during the first period. The mean Cl level was 105.9 (SE 0.14) mmol/L at baseline and decreased significantly by 0.54 (SD 0.14) mmol/L per 6 hours during the second period, and there were no significant changes during the first period. Lactate, Ca, K, and albumin-corrected calcium levels did not significantly change during the study. Cortisol levels also did not change significantly. However, cortisol levels appeared to be affected by long gaming sessions and a lack of rest during the study. A deviation from this pattern is usually associated with daily fluctuations in cortisol levels (Figure 3). During a normal cortisol rhythm, cortisol levels are at their lowest level at midnight before increasing to their peak value at 6 AM, followed by a steady decline toward midnight.

### Lipid Metabolism

Total cholesterol, LDL, high-density lipoprotein, and TG did not significantly change during the study. However, the TG levels during the study showed a distinct pattern: TG levels increased after evening meals but returned to baseline after 6 hours (Figure 4).

### Organ-Specific Markers

The mean ALP level was 77.2 (SE 0.63) U/L at baseline and increased during the second period by 3.8 (SD 0.63) U/L per 6 hours. There was no significant change during the first period. The mean bilirubin level was 9.9 (SE 0.22) μmol/L at baseline, decreased by 1.15 (SD 0.22) μmol/L per 6 hours during the first period and decreased by 1.05 (SD 0.22) μmol/L per 6 hours during the second period. The mean creatinine level was 93.6 (0.56) μmol/L at baseline, decreased by 2.9 (SD 0.56) μmol/L every 6 hours during the first period and decreased by 1.15 (SD 0.56) μmol/L every 6 hours during the second period. Albumin, CRP, ferritin, and ALT did not significantly change during the gaming sessions.

### Acid-Base Balance and Blood Gases

sO_2_, pCO_2_, pO_2_, pH, and standard bicarbonate concentration did not significantly change during the study.

### Hematology

The mean Hb level was 9.18 (SE 0.025) mmol/L at baseline, and it decreased by 0.055 (SD 0.025) mmol/L every 6 hours during the first period. However, during the second period, Hb increased by 0.027 (SD 0.025) mmol/L per 6 hours. The mean platelet count was 258×10^9^/L (SE 1.1×10^9^/L) at baseline and increased by 6.9×10^9^/L (SD 1.1×10^9^/L) per 6 hours during the second period. During the first session, there were no significant changes. Erythrocytes, EVF, mean cell Hb, and mean cell volume did not significantly change during the study.

## Discussion

### Principal Findings

This study is the first of its kind regarding the design and number of biochemical analyses in the gaming population. The fact that the study stretches over 42 hours of continuous measurements makes it unique compared to the current literature on gaming science. The study applies to recreational gamers who play with various levels of seriousness but lack a singular focus on the competition associated with e-sports. Overall, the effect of gaming on standard biochemical parameters in healthy male adults is limited. Significant changes were found in 12 of the 51 parameters. Most of the results of biochemical tests are novel findings in a gamer population [13]. It is not surprising that most parameters were unaffected by the intervention, but the high number of examined parameters added to our understanding of the effects of gaming. The participants had a large intake of calories throughout the gaming sessions (especially during the first gaming sessions) [14]. We found small changes in the levels of several biochemical and hematological biomarkers, but all the levels were within the normal range. Overall, the results of this exploratory study suggest that, from a biochemical and hematological standpoint, the health of male adults is not altered in the short term by long gaming sessions.

### Dehydration

The development of several parameters, including albumin, ALP, creatinine, Ca, ferritin, Hb, erythrocytes, EVF, K, and platelets, over time suggested that the participants were relatively dehydrated at the start of the study and rehydrated within the first 6 hours, followed by relative dehydration during the remainder of the study (Figure 1). Dehydration, despite a 3-L fluid intake per 18-hour gaming session, was most likely aided by the participants’ caffeine intake, as a large intake of caffeine during nonstrenuous activities can cause increased diuresis [25].

### Homeostasis

Blood glucose levels increased consistently during each of the gaming sessions (Figure 2). This development agrees with the findings of Chaput et al [15], but the changes in this study occurred over a much longer period. The changes in blood glucose levels cannot be attributed to dehydration, as this pathway is under tight hormonal control [26]. The glucose levels during the second session increased even though the participants had little energy intake. This could be due to the unexpectedly high levels of cortisol present at the same time (Figure 3), triggering the release of glucose from body stores. The glucose levels never exceeded the normal range and returned to baseline levels 1 week after the second long gaming session. Short- and long-term changes in blood glucose levels in gamers who regularly participate in long gaming sessions need further investigation.

The development of cortisol during the study differed from what would be expected (Figure 3). Cortisol is typically at its lowest level at midnight and increases sharply at 6 AM to reach a maximum between 6 and 10 AM, after which a sharp decline is expected [27]. The continued increase at noon during both sessions is surprising. Typically, the cortisol concentration decreases throughout the day and evening, reaching its nadir at midnight. The participants slept between noon (after the first session) and 6 PM. The 6 PM cortisol value could have been influenced by the waking cortisol response, which is related to the circadian rhythm [28]. Multiple factors potentially contribute to the disruption of the regular cortisol rhythm. Going into the study, the participants all adhered to a structured sleep pattern due to daytime employment or attending university classes. During both gaming sessions, we observed a rise in cortisol levels from 6 AM until noon. Gaming could be the cause of this change, as the level of alertness (or “stress”) in gaming potentially requires the continuous activity of the hypothalamic-pituitary-adrenal axis [28,29]. An increase in cortisol based on light intensity has been described, and light from monitors could be a factor in the changes observed [30]. Nightshift workers who are habitually awake during the night exhibit markedly lower morning cortisol levels [31]. The sharp decrease in cortisol levels at midnight (both nights) indicated that the regular diurnal slope was not completely changed by the weekend during extreme gaming.

### Lipid Metabolism

Gaming and sedentary behavior have been associated with obesity and increased cholesterol and TG levels [4], as they displace other nonsedentary activities [32]. Based on standard biochemistry, cholesterol and TG were unaffected by long gaming sessions and unrestricted food intake. However, TG levels sharply increased after mealtimes, which were normalized 6-12 hours after each meal. This increase was much greater than usual [33,34]. The sharp increase in TG was most likely the result of the high fat content of the ingested foods [14].

### Organ-Specific Markers

Overall, this study does not indicate that long gaming sessions impact the kidneys or livers of gamers. Creatinine levels decreased slightly during the study, possibly because of reduced physical activity. A decrease in creatinine at this level is not known to influence health.

Bilirubin and ALP levels decreased significantly during the study. This decrease, while significant, is not associated with any known pathophysiology.

Additionally, the large amounts of caffeine ingested may have lowered the bilirubin levels [35]. In experimental models, caffeine has been shown to have antifibrogenic, anti-inflammatory, and antioxidant effects that potentially exert liver protection [36-39].

We expected an increase in ALT based on food consumption during the study [40]. Likewise, we suspected that the inflammatory parameters would change due to the large intake of unhealthy food and drinks. As CRP, ferritin, and ALT are sensitive to changes in behavior (strenuous exercise, alcohol consumption, and excessive food intake), their lack of change during the study indicates a minor or no impact of gaming behavior.

### Acid-Base Balance and Blood Gases

Our results showed that gaming did not affect the pH balance or its regulatory systems. Furthermore, the pO_2_ in the venous blood did not significantly change throughout the trial. This finding agrees with the literature suggesting that sedentary gaming is not physically demanding [13].

### Hematology

Hematological parameters have not previously been examined in gamers during long gaming sessions. The Hb concentration decreased significantly during the first 18 hours of gaming and increased during the second 18-hour session. These changes are in accordance with the changes in hydration status outlined earlier. Thrombocyte counts increased throughout the study, especially in the second 18-hour session. Despite the significant changes, the parameters were always within the normal range. Overall, this exploratory study does not suggest a need for further investigation into the association between hematological parameters and gaming.

### Strengths and Limitations

The participants were all male individuals in their 20s, and there were only 9 participants. Based on these results, it is not possible to determine whether gaming has a different or even harmful effect on children, adolescents, or female individuals. The study was conducted in a hospital and not in the familiar environments of the participants. This may have caused participants to alter their behavior in ways we cannot determine. The easy availability of food and snacks may not relate entirely to how long gaming sessions are conducted at home or at local area network parties. We cannot rule out the possibility that overeating occurred, but the available food and drinks were only present at the behest of the participants. During the first 6-12 hours of the study, overeating was perhaps a factor, but during the second session, the participants had a markedly lower intake of calories from both food and drinks [14]. On the basis of this study, we are not able to determine whether the excessive intake of calories was due to convenience or to sustain a high level of performance during strenuous gaming.

### Conclusions

This study is the first of its kind, and many of the analyses in the study yielded novel results. The study was designed to emulate the behavior of gamers during the weekend and other long gaming sessions. At this point, we are not able to determine the difference between the effects of gaming and behavior during gaming. Regardless, the results of this study suggest that healthy gamers can partake in long gaming sessions, with ample amounts of unhealthy foods and little rest, without acute impacts on health.

## Appendix

Multimedia Appendix 1 CONSORT checklist.

Multimedia Appendix 2 Biochemical markers by analysis instrument.

Multimedia Appendix 3 Overview of biochemical markers.

## Abbreviations

ALP: alkaline phosphatase
ALT: alanine aminotransferase
Ca: calcium
Cl: chloride
CONSORT: Consolidated Standards of Reporting Trials
CRP: C-reactive protein
EVF: erythrocyte volume fraction
Hb: hemoglobin
K: potassium
LDL: low-density lipoprotein
Na: sodium
TG: triglyceride

## Data Availability

Data from this study are not available in a public repository. It can be requested from the corresponding author. The study garnered national media attention in Denmark when it was conducted, and several participants were interviewed through radio and television. The data included physical characteristics, employment status, and other personal information. Due to the low number of participants, it would potentially be possible to identify individual participants’ data.
