# Supplementary material for: Biochemical Changes in Adult Male Gamers During Prolonged Gaming: Pilot Study
Source: Interact J Med Res. 2024 Jul 8;13:e46570. doi: 10.2196/46570 (PMC11263886; doi:10.2196/46570)
Supplement: Multimedia Appendix 2 [file ijmr_v13i1e46570_app2.docx]

**Cobas 8000:** Albumin (Alb, g/L), Alanine Aminotransferase (ALT, U/L), Alkaline Phosphatase (Alp, U/L), Bilirubin (Bili, µmol/l), C-Reactive Protein (CRP, mg/L), Calcium (Ca, mmol/L), Calcium Albumin-Corrected (CaAC, mmol/L), Cortisol (Cor, nmol/L), Creatinine (Crea, μmol/l), Ferritin (Ftin, µg/l), Glucose (Glu, mmol/L), Potassium (K, mmol/L), Sodium (Na, mmol/L), Venous Blood Lactate (VBL, mmol/L), LDL Cholesterol (LDL, mmol/L), HDL Cholesterol (HDL, mmol/L), Triglycerides (TG, mmol/L).

**Sysmex XN-9000:** Basophils (Baso 10^9^/L), Eosinophils (Eos, 10^9^/L), Erythrocytes (Erc, 10^12^/L), Erythrocyte Volume Fraction (EVF), Hemoglobin (Hb, mmol/L), Hemoglobin content in reticulocytes (RetHb), Immature Reticulocyte Fraction (IRF), Intermediate Granulocytes (IMG), Leucocytes (Lcs, 10^9^/L), Lymphocytes (Lymph, 10^9^/L), Mean Cell Hemoglobin (MCH, 10^-15^ mol), Mean Cell Volume (MCV, 10^-15^ L), Mean Corpuscular Hemoglobin Concentration (MCHC, mmol/L), Mean Thrombocyte Volume (MPV, 10^-15^ L), Monocytes (Mono, 10^9^/L), Neutrophils (Neu, 10^9^/L), Red Blood Cell Distribution Width (RDW-SD, 10^-15^ mol), Relative Red Cell Distribution Width (RDW-CV), Reticulocytes (Reti, 10^9^/L), Thrombocyte Distribution Width (PDW-SD, 10^-15^ L), Thrombocytes (Trc, 10^9^/L), Thrombocytes Containing RNA (IPC, 10^9^/L).

**ABL800 FLEX:** Calcium (Ca, mmol/L), Carboxyhemoglobin (COHb, mmol/L), Chloride (Cl, mmol/L), Creatinine (Crea, µmol/L), Glucose (Glu, mmol/L), Lactate (Lac, mmol/L), Methemoglobin (MetHb, %), Oxyhemoglobin (HbO2 , %), Oxygen Saturation (sO2, %), Partial Pressure of Carbon Dioxide (pCO2, kPa), Partial Pressure of Oxygen (pO2, kPa), pH, Potassium (K, mmol/L), Sodium (Na, mmol/L), Standard Bicarbonate Concentration (SBC, mmol/L), Total Bilirubin (tBil, µmol/L), Total Hemoglobin (tHb, mmol/L).
