# Supplementary material for: Biochemical Changes in Adult Male Gamers During Prolonged Gaming: Pilot Study
Source: Interact J Med Res. 2024 Jul 8;13:e46570. doi: 10.2196/46570 (PMC11263886; doi:10.2196/46570)
Supplement: Multimedia Appendix 3 [file ijmr_v13i1e46570_app3.docx]

| Parameter | Baseline mean | Unit | Periode | Coefficient (slope) | SE | Lower CI | Upper CI | P Value | Visual test passed (Y/N) |
| --- | --- | --- | --- | --- | --- | --- | --- | --- | --- |
| Alanine Transaminase (ALT) | 23.90 | U/L | 1 | -0.28 | 0.28 | -0.8288 | 0.2688 | 5.1 | Yes |
|  |  |  | 2 | 0.19 | 0.28 | -0.3588 | 0.7388 |  |  |
| Albumin (Alb) | 45.3 | g/L | 1 | 0.3 | 0.17 | -0.0332 | 0.6332 | .051 | Yes |
|  |  |  | 2 | 0.49 | 0.17 | 0.1568 | 0.8232 |  |  |
| Alkaline Phosphatase (ALP) | 77.2 | U/L | 1 | 1.2 | 0.63 | -0.0348 | 24.348 | <.001 | Yes |
|  |  |  | 2 | 3.8 | 0.63 | 25.652 | 50.348 |  |  |
| Aspartate Transaminasse (AST) | 28.2 | U/L | 1 | -0.6 | 0.44 | -1.462 | 0.2624 | .02 | Yes |
|  |  |  | 2 | -1.2 | 0.44 | -2.062 | -0.3676 |  |  |
| Basophils (Baso) | 0.05 | 10^9^/L | 1 | -0.00024 | 0.0011 | -0.0024 | 0.001916 | 27.5 | Yes |
|  |  |  | 2 | 0.00069 | 0.0011 | -0.00147 | 0.002846 |  |  |
| Bilirubin (Bili) | 9.9 | µmol/L | 1 | -1.15 | 0.22 | -15.812 | -0.7188 | <.001 | Yes |
|  |  |  | 2 | -1.05 | 0.22 | -14.812 | -0.6188 |  |  |
| Calcium (Ca) | 2.41 | mmol/L | 1 | 0.011 | 0.0057 | -0.00017 | 0.022172 | 1.4 | Yes |
|  |  |  | 2 | 0.016 | 0.0057 | 0.004828 | 0.027172 |  |  |
| Calcium Albumin-Corrected (CaAC) | 2.34 | mmol/L | 1 | 0.013 | 0.0044 | 0.004376 | 0.021624 | .82 | Yes |
|  |  |  | 2 | 0.012 | 0.0044 | 0.003376 | 0.020624 |  |  |
| Carboxyhemoglobin (COHb) | 0.03 | mmol/L | 1 | -0.00076 | 0.0006 | -0.00194 | 0.000416 | .35 | Yes |
|  |  |  | 2 | -0.00023 | 0.0006 | -0.00141 | 0.000946 |  |  |
| Chloride (Cl) | 105.90 | mmol/L | 1 | -0.19 | 0.14 | -0.4644 | 0.0844 | <.001 | Yes |
|  |  |  | 2 | -0.54 | 0.14 | -0.8144 | -0.2656 |  |  |
| Cholesterol (Chol) | 4 | mmol/L | 1 | -0.068 | 0.019 | -0.10524 | -0.03076 | .12 | Yes |
|  |  |  | 2 | -0.041 | 0.019 | -0.07824 | -0.00376 |  |  |
| Cortisol (Cor) | 209.92 | nmol/L | 1 | 22.44 | 15.55 | -8.038 | 52.918 | 13.3 | Yes |
|  |  |  | 2 | 25.03 | 15.55 | -5.448 | 55.508 |  |  |
| C-Reactive Protein (CRP) | 1.43 | mg/L | 1 | 0.0069 | 0.043 | -0.07738 | 0.09118 | 47.9 | Yes |
|  |  |  | 2 | -0.0041 | 0.043 | -0.08838 | 0.08018 |  |  |
| Creatinine (Crea) | 93.6 | µmol/L | 1 | -2.916 | 0.56 | -40.136 | -18.184 | <.001 | Yes |
|  |  |  | 2 | -1.15 | 0.56 | -22.476 | -0.0524 |  |  |
| Eosinophils (Eos) | 0.21 | 10^9^/L | 1 | -0.01 | 0.007 | -0.02372 | 0.00372 | 8.7 | Yes |
|  |  |  | 2 | -0.0013 | 0.007 | -0.01502 | 0.01242 |  |  |
| Erythrocyte Volume Fraction (EVF) | 0.43 |  | 1 | -0.0023 | 0.0012 | -0.00465 | 0.000052 | .07 | Yes |
|  |  |  | 2 | 0.0011 | 0.0012 | -0.00125 | 0.003452 |  |  |
| Erythrocytes (Erc) | 5.04 | 10^12^/L | 1 | -0.025 | 0.013 | -0.05048 | 0.00048 | .2 | Yes |
|  |  |  | 2 | 0.01 | 0.013 | -0.01548 | 0.03548 |  |  |
| Ferritin (Ftin) | 156 | µg/L | 1 | -1.91 | 0.76 | -33.996 | -0.4204 | 2.5 | Yes |
|  |  |  | 2 | -1.33 | 0.76 | -28.196 | 0.1596 |  |  |
| Glucose (Glu) | 4.39 | mmol/L | 1 | 0.24 | 0.07 | 0.1028 | 0.3772 | <.001 | Yes |
|  |  |  | 2 | 0.38 | 0.07 | 0.2428 | 0.5172 |  |  |
| HDL Cholesterol (HDL) | 1.2 | mmol/L | 1 | 0.0084 | 0.0093 | -0.00983 | 0.026628 | 6.6 | Yes |
|  |  |  | 2 | 0.018 | 0.0093 | -0.00023 | 0.036228 |  |  |
| Hemoglobin (Hb) | 9.18 | mmol/L | 1 | -0.055 | 0.025 | -0.104 | -0.006 | .01 | Yes |
|  |  |  | 2 | 0.027 | 0.025 | -0.022 | 0.076 |  |  |
| Immature Reticulocyte Fraction (IRF) | 0.06 |  | 1 | 0.0012 | 0.0013 | -0.00135 | 0.003748 | .004 | Yes |
|  |  |  | 2 | 0.0051 | 0.0013 | 0.002552 | 0.007648 |  |  |
| Intermediate Granulocytes (IMG) | 0.026 |  | 1 | 0.0023 | 0.0011 | 0.000144 | 0.004456 | 6.6 | Yes |
|  |  |  | 2 | 0.0012 | 0.0011 | -0.00096 | 0.003356 |  |  |
| Lactate (Lac) | 1.30 | mmol/L | 1 | 0.14 | 0.04 | 0.0616 | 0.2184 | .002 | Yes |
|  |  |  | 2 | 0.13 | 0.04 | 0.0516 | 0.2084 |  |  |
| LDL Cholesterol | 2.14 | mmol/L | 1 | -0.045 | 0.026 | -0.09596 | 0.00596 | .2 | Yes |
|  |  |  | 2 | -0.086 | 0.026 | -0.13696 | -0.03504 |  |  |
| Leucocytes (Lcs) | 7 | 10^9^/L | 1 | 0.0061 | 0.068 | -0.12718 | 0.13938 | - | Yes |
|  |  |  | 2 | 0.0055 | 0.068 | -0.12778 | 0.13878 |  |  |
| Lymphocytes (Lymph) | 2.34 | 10^9^/L | 1 | -0.055 | 0.051 | -0.15496 | 0.04496 | 25.0 | Yes |
|  |  |  | 2 | -0.02 | 0.051 | -0.11996 | 0.07996 |  |  |
| Mean Cell Hemoglobin (MCH) | 1.8 | 10^-15^ mol | 1 | -0.002 | 0.0018 | -0.00553 | 0.001528 | 2.1 | Yes |
|  |  |  | 2 | 0.0017 | 0.0018 | -0.00183 | 0.005228 |  |  |
| Mean Cell Volume (MCV) | 85.9 | 10^-15^ mol | 1 | 0.058 | 0.088 | -0.11448 | 0.23048 | 31.1 | Yes |
|  |  |  | 2 | 0.087 | 0.088 | -0.08548 | 0.25948 |  |  |
| Mean Corpuscular Hemoglobin Concentration (MCHC) | 21.2 | mmol/L | 1 | -0.03 | 0.031 | -0.09076 | 0.03076 | 13.3 | Yes |
|  |  |  | 2 | 0.009 | 0.031 | -0.05176 | 0.06976 |  |  |
| Mean Thrombocyte Volume (MPV) | 10.6 | 10^-15^ L | 1 | -0.037 | 0.019 | -0.07424 | 0.00024 | 5.1 | Yes |
|  |  |  | 2 | -0.015 | 0.019 | -0.05224 | 0.02224 |  |  |
| Methemoglobin (MetHb) | 0.01 | % | 1 | -0.00033 | 0.00011 | -0.00055 | -0.00011 | .01 | Yes |
|  |  |  | 2 | -0.00015 | 0.00011 | -0.00037 | 6.56E-05 |  |  |
| Monocytes (Mono) | 0.61 | 10^9^/L | 1 | 0.007 | 0.009 | -0.01064 | 0.02464 | 7.7 | Yes |
|  |  |  | 2 | -0.007 | 0.009 | -0.02464 | 0.01064 |  |  |
| Neutrophils (Neu) | 3.7 | 10^9^/L | 1 | 0.062 | 0.06 | -0.0556 | 0.1796 | 28.1 | Yes |
|  |  |  | 2 | 0.031 | 0.06 | -0.0866 | 0.1486 |  |  |
| Oxygen Saturation (SO2) | 0.97 | % | 1 | -0.37 | 0.015 | -0.3994 | -0.3406 | .051 | No |
|  |  |  | 2 | -0.027 | 0.015 | -0.0564 | 0.0024 |  |  |
| Oxyhemoglobin (HbO2) | 0.93 | % | 1 | -0.034 | 0.014 | -0.06144 | -0.00656 | .052 | No |
|  |  |  | 2 | -0.026 | 0.014 | -0.05344 | 0.00144 |  |  |
| Partial Pressure of Carbon Dioxide (pCO2) | 5.90 | kPa | 1 | 0.0065 | 0.074 | -0.13854 | 0.15154 | .3 | Yes |
|  |  |  | 2 | -0.08 | 0.074 | -0.22504 | 0.06504 |  |  |
| Partial Pressure of Oxygen (pO2) | 17.73 | kPa | 1 | -1,78 | 0.83 | -34.068 | -0.1532 | .1 | Yes |
|  |  |  | 2 | -1,48 | 0.83 | -31.068 | 0.1468 |  |  |
| pH | 7.37 |  | 1 | 0.0003 | 0.0035 | -0.00656 | 0.00716 | .1 | Yes |
|  |  |  | 2 | 0.0055 | 0.0035 | -0.00136 | 0.01236 |  |  |
| Potassium (K) | 3.8 | mmol/L | 1 | -0.048 | 0.026 | -0.09896 | 0.00296 | 9.7 | Yes |
|  |  |  | 2 | -0.036 | 0.026 | -0.08696 | 0.01496 |  |  |
| Red Blood Cell Distribution Width (RDW-SD) | 38.32 | 10^-15^ mol | 1 | -0.029 | 0.062 | -0.15052 | 0.09252 | 43.9 | Yes |
|  |  |  | 2 | -0.0087 | 0.062 | -0.13022 | 0.11282 |  |  |
| Relative Red Cell Distribution Width (RDW-CV) | 0.12 |  | 1 | -0.00024 | 0.00036 | -0.00095 | 0.000466 | 30.4 | Yes |
|  |  |  | 2 | 0.000019 | 0.00036 | -0.00069 | 0.000725 |  |  |
| Reticulocyte hemoglobin Content (Rethb) | 2.07 | fmol | 1 | 0.0038 | 0.0035 | -0.00306 | 0.01066 | .1 | Yes |
|  |  |  | 2 | -0.006 | 0.0035 | -0.01286 | 0.00086 |  |  |
| Reticulocytes (Reti) | 68.9 | 10^9^/L | 1 | -0.56 | 0.49 | -15.204 | 0.4004 | 12.2 | Yes |
|  |  |  | 2 | 0.043 | 0.49 | -0.9174 | 10.034 |  |  |
| Sodium (Na) | 143.1 | mmol/L | 1 | -0.1 | 0.12 | -0.3352 | 0.1352 | .002 | Yes |
|  |  |  | 2 | -0.48 | 0.12 | -0.7152 | -0.2448 |  |  |
| Standard Bicarbonate Concentration (SBC) | 24.41 | mmol/L | 1 | -0.022 | 0.058 | -0.13568 | 0.09168 | .2 | Yes |
|  |  |  | 2 | 0.058 | 0.058 | -0.05568 | 0.17168 |  |  |
| Thrombocyte Distribution Width (PDW-SD) | 12.49 | 10^-15^ L | 1 | -0.064 | 0.057 | -0.17572 | 0.04772 | 13.3 | Yes |
|  |  |  | 2 | 0.0028 | 0.057 | -0.10892 | 0.11452 |  |  |
| Thrombocytes (Trc) | 258 | 10^9^/L | 1 | 0.65 | 1.1 | -1.506 | 2.806 | <.001 | Yes |
|  |  |  | 2 | 6.9 | 1.1 | 4.744 | 9.056 |  |  |
| Thrombocytes Containing RNA (IPC) | 11.1 |  | 1 | 0.014 | 0.2 | -0.378 | 0.406 | 32.1 | Yes |
|  |  |  | 2 | 0.14 | 0.2 | -0.252 | 0.532 |  |  |
| Total Bilirubin (tBil) | 15.00 | µmol/L | 1 | -1.57 | 0.49 | -25.304 | -0.6096 | .007 | Yes |
|  |  |  | 2 | -1.33 | 0.49 | -22.904 | -0.3696 |  |  |
| Total Hemoglobin (tHb) | 9.61 | mmol/L | 1 | -0.072 | 0.024 | -0.11904 | -0.02496 | <.001 | Yes |
|  |  |  | 2 | 0.024 | 0.024 | -0.02304 | 0.07104 |  |  |
| Triglyceride Non-Fractionated (Tgly-NF) | 1.55 | mmol/L | 1 | -0.089 | 0.093 | -0.27128 | 0.09328 | 6.1 | Yes |
|  |  |  | 2 | 0.059 | 0.093 | -0.12328 | 0.24128 |  |  |
| Venous Blood Lactate (VBL) | 1.3 | mmol/L | 1 | 0.14 | 0.04 | 0.0616 | 0.2184 | .1 | Yes |
|  |  |  | 2 | 0.14 | 0.04 | 0.0616 | 0.2184 |  |  |
|  |  |  |  |  |  |  |  |  |  |
